# MASH-GA: a manually curated cross-species transcriptomic database for metabolic-associated steatohepatitis

**DOI:** 10.1093/database/baag013

**Published:** 2026-04-02

**Authors:** Yong Wang, Ke Ding, Beicheng Sun, Qingxiang Xu, Weiting Zheng, Lei Liu, Jian Wang, Kanghui Liu, Jinyao Zhang, Wenjie Zhang

**Affiliations:** Department of Hepatobiliary Surgery, The First Affiliated Hospital of Anhui Medical University, Hefei, Anhui, China; MOE Innovation Center for Basic Research in Tumor Immunotherapy, Hefei, Anhui, China; Anhui Province Key Laboratory of Tumor Immune Microenvironment and Immunotherapy, Hefei, Anhui, China; Department of Hepatobiliary Surgery, The First Affiliated Hospital of Anhui Medical University, Hefei, Anhui, China; MOE Innovation Center for Basic Research in Tumor Immunotherapy, Hefei, Anhui, China; Anhui Province Key Laboratory of Tumor Immune Microenvironment and Immunotherapy, Hefei, Anhui, China; Department of Hepatobiliary Surgery, The First Affiliated Hospital of Anhui Medical University, Hefei, Anhui, China; MOE Innovation Center for Basic Research in Tumor Immunotherapy, Hefei, Anhui, China; Anhui Province Key Laboratory of Tumor Immune Microenvironment and Immunotherapy, Hefei, Anhui, China; Department of Hepatobiliary Surgery, The First Affiliated Hospital of Anhui Medical University, Hefei, Anhui, China; MOE Innovation Center for Basic Research in Tumor Immunotherapy, Hefei, Anhui, China; Anhui Province Key Laboratory of Tumor Immune Microenvironment and Immunotherapy, Hefei, Anhui, China; Department of Hepatobiliary Surgery, The First Affiliated Hospital of Anhui Medical University, Hefei, Anhui, China; MOE Innovation Center for Basic Research in Tumor Immunotherapy, Hefei, Anhui, China; Anhui Province Key Laboratory of Tumor Immune Microenvironment and Immunotherapy, Hefei, Anhui, China; Department of Hepatobiliary Surgery, The First Affiliated Hospital of Anhui Medical University, Hefei, Anhui, China; MOE Innovation Center for Basic Research in Tumor Immunotherapy, Hefei, Anhui, China; Anhui Province Key Laboratory of Tumor Immune Microenvironment and Immunotherapy, Hefei, Anhui, China; Department of Hepatobiliary Surgery, The First Affiliated Hospital of Anhui Medical University, Hefei, Anhui, China; MOE Innovation Center for Basic Research in Tumor Immunotherapy, Hefei, Anhui, China; Anhui Province Key Laboratory of Tumor Immune Microenvironment and Immunotherapy, Hefei, Anhui, China; Department of Hepatobiliary Surgery, The First Affiliated Hospital of Anhui Medical University, Hefei, Anhui, China; MOE Innovation Center for Basic Research in Tumor Immunotherapy, Hefei, Anhui, China; Anhui Province Key Laboratory of Tumor Immune Microenvironment and Immunotherapy, Hefei, Anhui, China; Department of Hepatobiliary Surgery, The First Affiliated Hospital of Anhui Medical University, Hefei, Anhui, China; MOE Innovation Center for Basic Research in Tumor Immunotherapy, Hefei, Anhui, China; Anhui Province Key Laboratory of Tumor Immune Microenvironment and Immunotherapy, Hefei, Anhui, China; Department of Hepatobiliary Surgery, The First Affiliated Hospital of Anhui Medical University, Hefei, Anhui, China; MOE Innovation Center for Basic Research in Tumor Immunotherapy, Hefei, Anhui, China; Anhui Province Key Laboratory of Tumor Immune Microenvironment and Immunotherapy, Hefei, Anhui, China

## Abstract

Metabolic dysfunction-associated steatohepatitis (MASH), formerly called NASH, is a progressive form of fatty liver disease closely linked to metabolic dysregulation. It has become a leading cause of liver failure and liver transplantation worldwide. Despite its growing clinical significance, there is currently no centralized, publicly accessible, cross-cohort and cross-species transcriptomic database specifically dedicated to MASH. To address this gap, we developed MASH-GA (MASH—Genomic Atlas; https://mashga.com), the first and only database in the fatty liver research field that systematically integrates transcriptomic data across human cohorts and mouse models. MASH-GA contains 45 human transcriptomic datasets (2740 samples) from 17 countries and 126 mouse datasets (1950 samples) covering major MASH models. The platform provides a range of unique features, including integrated weighted gene coexpression network analysis (WGCNA), cross-model classification and comparison of mouse datasets, multigene correlation analysis and pairwise coexpression exploration, and interactive visualizations (e.g. boxplots and correlation plots). Human and mouse data are presented in separate, intuitively navigable modules. As the only standardized, multicohort, cross-species MASH transcriptomic database currently available, MASH-GA enables reproducible data exploration, informed model selection, and the identification of regulatory modules. It is therefore a valuable resource for researchers in hepatology, metabolic disease, and systems biology. **Database URL:**  https://mashga.com

## Introduction

Metabolic dysfunction-associated steatohepatitis (MASH), formerly known as nonalcoholic steatohepatitis (NASH), is a chronic liver disease closely associated with obesity, type 2 diabetes, and insulin resistance [1–3]. It is characterized by hepatic steatosis, ballooning degeneration of hepatocytes, and inflammatory infiltration. Among fatty liver disease subtypes, MASH represents the most progressive form, affecting over 100 million individuals worldwide and continuing to rise in prevalence [4, 5]. It can eventually lead to cirrhosis and hepatocellular carcinoma [6]. Despite numerous drug candidates entering clinical trials, no approved targeted therapy is currently available, underscoring the urgent need to identify novel therapeutic targets and biomarkers [7].

With the advancement of transcriptomics and other omics technologies, large-scale high-throughput data have been widely used to dissect the molecular mechanisms of MASH. Most transcriptomic datasets are scattered across public repositories such as the Gene Expression Omnibus (GEO) [8]. Although existing tools, including GREIN [9], ImaGEO [10], and eVITTA [11], enable reanalysis and visualization of individual datasets, several important limitations remain for disease-focused and translational research. First, these platforms generally rely on heterogeneous and sparsely annotated metadata, making it difficult to perform refined queries based on combined criteria such as disease stage, experimental model, and treatment condition. Second, the lack of standardized processing and harmonization across studies limits direct cross-cohort comparisons of specific genes or pathways, thereby constraining biomarker validation and generalizability. Third, support for systems-level analyses, such as coexpression network exploration, is limited, reducing the ability to derive mechanistic insights from integrated datasets. Finally, none of the existing resources provides a unified framework for standardized integration of transcriptomic data across both species and cohorts, which is essential for translational model selection in MASH research.

In addition, various diet-induced mouse models (e.g. high-fat diet [HFD], choline-deficient HFD [CDAHFD], methionine–choline-deficient diet [MCD], streptozotocin-induced diabetic fatty liver model [STAM], high-fat, high-sucrose, high-cholesterol diet [HFHSHC], and Western diet combined with carbon tetrachloride [WD–CCl₄] administration) are widely used to simulate different stages of human MASH [12]. However, these models exhibit substantial heterogeneity in disease progression, particularly in terms of inflammation, metabolic disturbance, and fibrosis severity [13–15]. Therefore, cross-model and cross-species data integration is crucial for selecting appropriate experimental models, elucidating disease mechanisms, and validating candidate targets.

Although murine models capture many transcriptomic hallmarks of MASH, fundamental species-level differences in intermediary metabolism warrant caution when extrapolating mechanistic findings—particularly for pathways governing lipid and glucose homeostasis. Mice have a basal metabolic rate per unit body mass roughly 7–15 times higher than that of humans, which drives a correspondingly greater basal glucose turnover; as a result, their fasting blood glucose is typically lower yet fluctuates much more rapidly [16]. Mice lack cholesteryl ester transfer protein (CETP) activity and therefore exhibit a high-density lipoprotein (HDL)-dominant plasma lipoprotein profile, whereas humans carry most cholesterol in low-density lipoprotein (LDL) particles [17]; this alters hepatic lipid flux and very-low-density lipoprotein triglyceride (VLDL-TG) secretion requirements. Basal hepatic *de novo* lipogenesis is also two- to three-fold higher in mice and is predominantly regulated by carbohydrate response element-binding protein (ChREBP) [18], while in humans sterol regulatory element-binding protein-1c (SREBP-1c) plays a larger role.

To address these challenges in transcriptomic analysis, we developed MASH-GA (MASH—Genomic Atlas, https://mashga.com), a manually curated, cross-species transcriptomic database specifically designed for MASH. MASH-GA features five key components: (i) a rigorously curated database integrating 45 human transcriptomic datasets (2740 samples) from 17 countries and 126 mouse datasets (1950 samples) representing major MASH models; (ii) a structured keyword-based query system that enables multiattribute filtering based on disease status, model type, treatment, timepoint, and tissue source; (iii) a suite of interactive analysis tools supporting coexpression module scoring, multigene visualization, and correlation analysis; (iv) capabilities for gene-level comparisons across models and cohorts, facilitating model selection and target validation; and (v) two case studies that demonstrate the unique value of MASH-GA in uncovering disease mechanisms and identifying candidate therapeutic targets.

## Materials and methods

### Dataset collection and metadata curation

**Figure 1:**
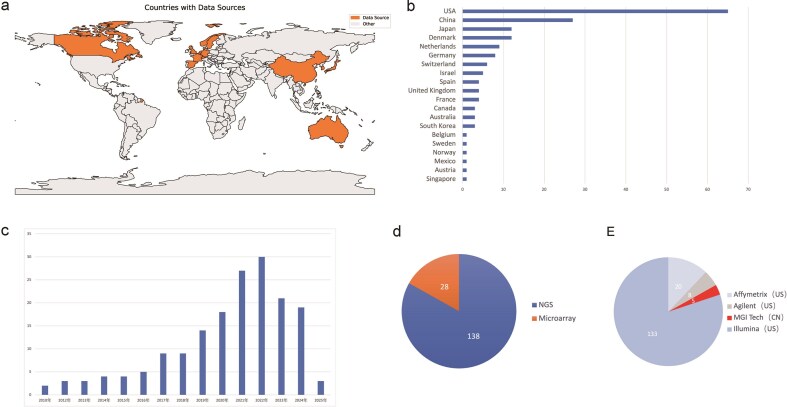
Overview of the MASH-GA dataset composition. (a) Countries contributing human datasets (*n* = 17). (b) Dataset counts per country. (c) Year-by-year accrual of studies (2010–2025 H1). (d) Proportion of NGS (*n* = 138) versus microarray (*n* = 28) datasets. (e) Distribution of sequencing platforms among NGS datasets.

MASH-GA comprises a total of 171 transcriptomic datasets, including 45 human datasets (2740 samples) derived from 17 countries (Fig. 1a–c) and 126 mouse datasets (1950 samples) representing major dietary-induced MASH models such as HFD, MCD, HFHS, HFHSHC, CDAHFD, and STAM.

All datasets were retrieved primarily through systematic searches of the GEO repository and include both microarray and RNA-seq studies (Fig. 1d and e), with the latter comprising the majority. For RNA-seq datasets, expression matrices were either obtained directly from the original studies or generated in-house from raw sequencing files when available. Sequencing quality was assessed using FastQC (v0.11.9). Reads were aligned to the reference genome using HISAT2 (v2.2.1), and gene-level quantification was performed with featureCounts (v2.0.3). Differential expression analysis was conducted in the R environment (v4.4.3) using DESeq2 (v1.46.0). For microarray datasets, raw probe-level intensities were background-corrected and normalized using array-specific standard procedures. Differential expression analysis was performed using the limma package (v3.62.2). Microarray probe IDs were mapped to standardized HUGO gene symbols with the biomaRt R package or platform-specific annotation files supplied by the array manufacturer; when multiple probes corresponded to the same gene, the median probe intensity was taken as the representative expression value. Differential gene expression was defined primarily based on statistical significance, using a false discovery rate (FDR) threshold of <0.05. Log2 fold change (logFC) values were reported in the analysis results to indicate effect size.

Each dataset was manually curated into a structured metadata scheme with five core attributes: (i) disease status (healthy, obese, NAFLD, and NASH), (ii) NAFLD activity score (NAS 0–8), (iii) fibrosis stage (F0–F4), (iv) species/model type (e.g. HFD, MCD, STAM, HFHS, HFHSHC, and CDAHFD), and (v) treatment or time point (when applicable). Every keyword was first normalized and then partially mapped to controlled vocabularies (MeSH, MedDRA LLT, and NCIT) to ensure semantic consistency and to optimize query performance. Most human samples derive from liver-biopsy tissue, whereas mouse datasets cover multiple experimental models with corresponding interventions and time-series data. Differential expression was precomputed with DESeq2 (RNA-seq) or limma (microarray), and all expression matrices, metadata, DEG tables, and coexpression module annotations were stored in a relational database to enable rapid, flexible querying.

### Data analysis modules

**Figure 2:**
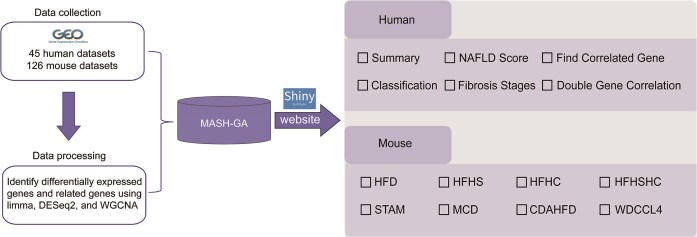
Overview of the MASH-GA pipeline and analysis modules. GEO datasets are curated into the MASH-GA database, which is served through an R Shiny web interface. The Human module offers six analytical views, whereas the Mouse module visualizes gene expression across eight NASH models.

MASH-GA provides two functionally independent and user-friendly modules—MASH-Human and MASH-Mouse—dedicated to the visualization, comparison, and integration of transcriptomic data from human clinical samples and mouse experimental models, respectively (Fig. 2).

### MASH-Human module

The MASH-Human module focuses on analysing gene expression patterns in liver tissue samples from MASH patients and their associations with clinical phenotypes such as disease status, inflammation score, and fibrosis stage. It includes several functional subpages that allow users to explore gene-specific patterns and potential regulatory associations from different perspectives:

Summary: Displays the overall expression distribution of a target gene across all human samples, stratified by disease status (e.g. healthy, NAFL, NAFLD, and NASH), to provide an initial overview of its expression trend.
Classification: Presents gene expression levels grouped by disease categories within each dataset, supporting cross-dataset consistency checks and model-based comparisons.
NAFLD Score: Enables sample grouping based on the NAFLD Activity Score (NAS, ranging from 0 to 8), facilitating the assessment of gene expression trends across different levels of disease activity, such as steatosis and inflammation.
Fibrosis Stages: Allows analysis of gene expression dynamics across fibrosis stages (F0–F4), helping identify candidate genes associated with fibrosis progression.
Multigene Analysis: This section is based on weighted gene coexpression network analysis (WGCNA) and supports the identification of genes coexpressed with the target gene. It includes two subfunctions: (i) Find Correlated Gene: Automatically calculates genome-wide expression correlations with the target gene and ranks the most associated genes, aiding in the discovery of putative regulatory modules. (ii) Double Gene Correlation: Allows users to input any two genes, plot a scatter plot of their expression values, and compute the Spearman correlation coefficient to visualize coexpression relationships.

### MASH-Mouse module

The MASH-Mouse module visualizes and compares hepatic gene-expression profiles across a wide spectrum of murine MASH models. After the user enters a target gene, the system automatically groups samples by induction protocol—HFD, HFHC, HFHS, HFHSHC, WD–CCl₄, CDAHFD, MCD, and the STAM model, which couples low-dose streptozotocin with an HFD—and then generates box plots to highlight expression differences among models. For datasets that include longitudinal sampling or pharmacological interventions, the module can further stratify results by time point (e.g. W0, W4, W8) or treatment condition (e.g. vehicle vs. drug), enabling dynamic tracking of gene-expression changes throughout disease progression.

## Results

### Fibrotic, inflammatory, and metabolic gene programmes in MASH

**Figure 3:**
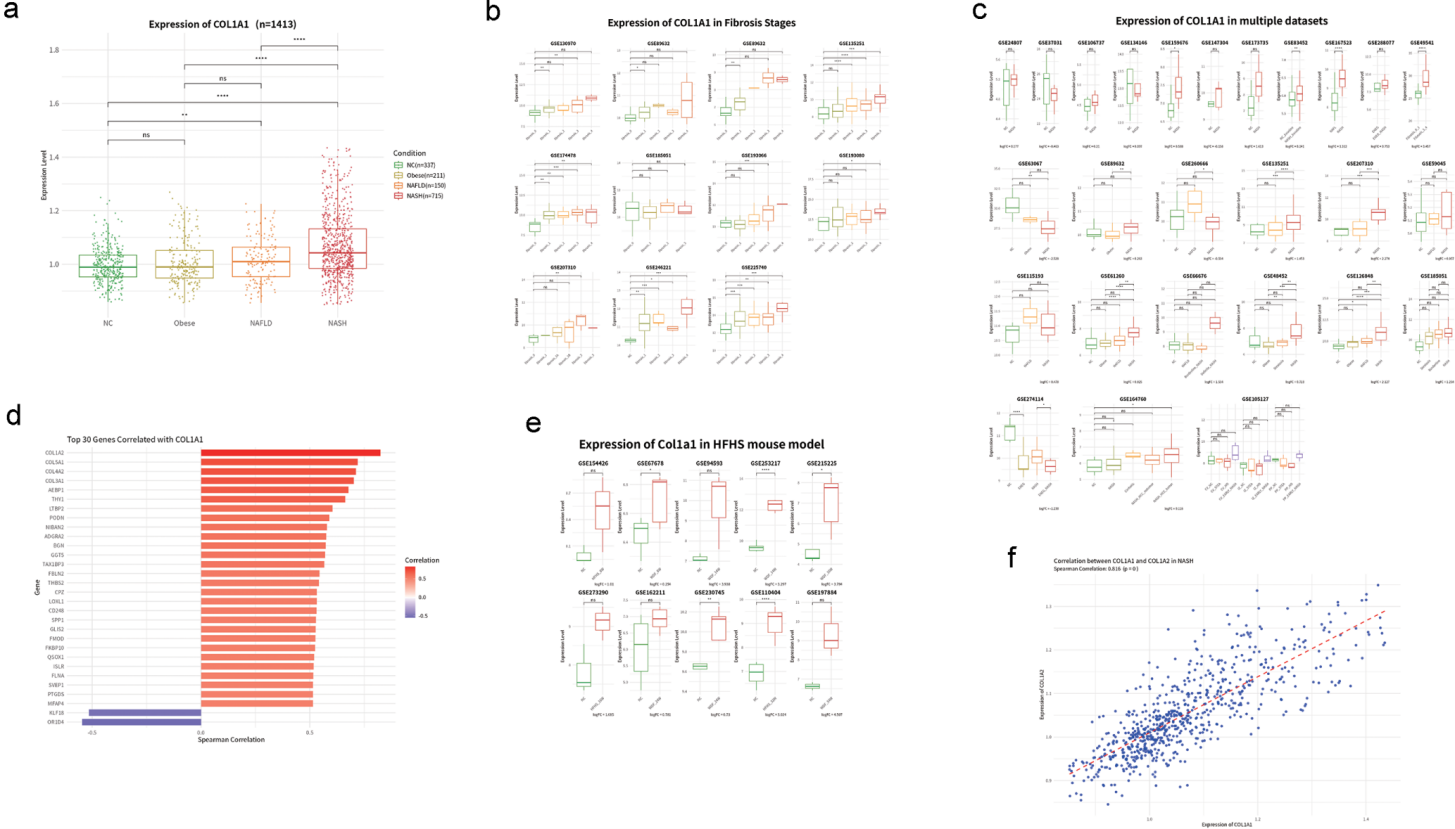
Multilevel assessment of COL1A1 expression and coexpression in MASH-GA. (a) COL1A1 increases stepwise from normal controls (NC) to obese and NAFLD and peaks in NASH (human cohort, *P* < .001). (b) Across 10 independent datasets, COL1A1 rises with fibrosis stage, F0 → F4. (c) Cross-cohort comparison confirms consistent upregulation of COL1A1 in NASH relative to earlier stages. (d) Top 30 genes most strongly coexpressed with COL1A1, highlighting an extracellular-matrix module. (e) Parallel induction of Col1a1 in HFHS mouse models mirrors the human pattern. (f) Gene-pair analysis shows tight coordination between COL1A1 and COL1A2 in NASH liver samples (Spearman’s *r* > 0.7).

COL1A1 (Collagen Type I Alpha 1 Chain), encoding the major structural component of type I collagen, is a well-established marker of liver fibrosis and is tightly associated with hepatic stellate cell activation and extracellular matrix (ECM) remodelling [19–21]. By leveraging the MASH–GA database, we systematically profiled the expression dynamics of COL1A1 across different stages of MASH, integrating data from clinical samples, fibrosis staging, cross-cohort validation, mouse models, and coexpression analyses (Fig. 3a–f). In human samples, COL1A1 expression progressively increased along the disease continuum from normal control (NC), obese, NAFLD to NASH, with a marked upregulation observed specifically at the NASH stage (*P* < .001), suggesting its central role in fibrotic remodelling. While expression remained unchanged in obese individuals, a mild elevation was noted in NAFLD, implying early ECM responses prior to overt fibrosis. Consistently, in multiple datasets with fibrosis grading, COL1A1 levels were significantly elevated in advanced stages (F3–F4) compared to early fibrosis (F0–F1), reinforcing its utility as a stage-discriminating biomarker. Cross-cohort validation across >20 independent transcriptomic studies confirmed the robust and reproducible upregulation of COL1A1 in NASH samples, regardless of population or platform differences. Network analysis revealed that COL1A1 was strongly coexpressed with key ECM genes such as COL1A2, COL5A1, and SPP1, forming a conserved fibrosis-related gene module. Correlation analysis further demonstrated that COL1A1 and COL1A2 exhibited a Spearman’s correlation coefficient exceeding 0.7, indicating close transcriptional coordination. Moreover, in HFHS diet-induced mouse models, COL1A1 expression showed consistent upregulation, paralleling human disease patterns and supporting its cross-species conservation. These findings collectively highlight COL1A1 as a robust molecular indicator of fibrotic progression in MASH and a reliable readout for both mechanistic exploration and therapeutic evaluation in preclinical models.

Tumour necrosis factor (TNF), a central pro-inflammatory cytokine, was examined as a representative marker of inflammatory activation in MASH. Across human cohorts, TNF expression showed minimal changes in obese samples but was progressively upregulated in NAFLD and further increased in NASH, consistent with the emergence and amplification of hepatic inflammatory responses during disease progression. These patterns were reproducible across multiple independent datasets, highlighting the ability of MASH-GA to capture inflammation-related transcriptional programmes in a stage-dependent manner. SREBF1 (sterol regulatory element-binding transcription factor 1), a key transcriptional regulator of *de novo* lipogenesis, was analysed to illustrate metabolic alterations during early disease stages. SREBF1 expression was elevated predominantly in obese and early NAFLD samples, reflecting metabolic reprogramming and enhanced lipid synthesis prior to advanced inflammatory and fibrotic remodelling. In later NASH stages, SREBF1 expression showed relative stabilization or attenuation, consistent with a shift from metabolic dysregulation towards inflammation- and fibrosis-dominated molecular programmes.

### Stage-wise transcriptomic validation confirms MASH-GA fidelity

To evaluate the biological fidelity of MASH-GA, we reanalysed RNA-seq and microarray datasets from the Human module with DESeq2 and limma (FDR < 0.05), focusing on three pivotal disease transitions—obese vs. normal, NAFLD vs. normal, and NASH vs. NAFLD—and performed GO enrichment with clusterProfiler.

Obese vs. normal revealed only modest transcriptional changes, with enriched GO terms such as ‘fatty-acid metabolic process’ and ‘response to oxidative stress’, mirroring the subtle metabolic remodelling reported for simple obesity.

NAFLD vs. normal showed a broader lipogenic signature coupled with mild pro-inflammatory signals; enriched terms included ‘fatty-acid biosynthetic process’, ‘steroid metabolic process’, and ‘response to inflammatory stimulus’.

NASH vs. NAFLD displayed the most pronounced shift, dominated by fibrotic remodelling; GO enrichment highlighted ‘extracellular-matrix organization’, a hallmark of late-stage MASH.

**Figure 4:**
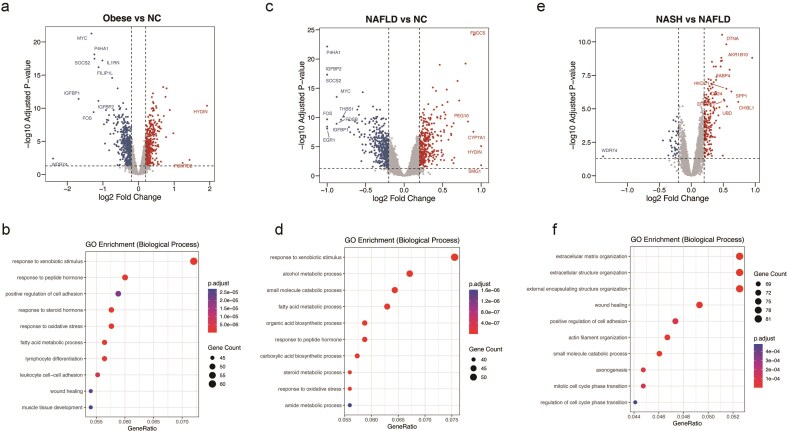
Differential expression and functional enrichment analyses across disease stages. (a, b) Differential expression volcano plot (a) and GO Biological Process enrichment bubble plot (b) for obese vs. normal. (c, d) Differential expression volcano plot (c) and GO Biological Process enrichment bubble plot (d) for NAFLD vs. normal. (e, f) Differential expression volcano plot (e) and GO Biological Process enrichment bubble plot (f) for NASH vs. NAFLD.

These stage-specific expression and functional changes are visualized in (Fig. 4a, c, and e) (volcano plots) and (Fig. 4b, d, and f) (GO bubble plot), and recapitulate established disease biology. These stage-specific patterns closely mirror the consensus trajectory reported in previous multiomics studies. In simple obesity, transcriptomic changes are largely confined to pathways governing lipid handling, beta-oxidation, and oxidative-stress buffering, with minimal immune engagement [22]. Early NAFLD intensifies *de novo* lipogenesis and steroid-biosynthetic programmes while triggering low-grade cytokine and chemokine signalling, consistent with hepatocellular lipid overload and metabolic inflammation [23]. The transition to NASH is accompanied by a marked shift towards extracellular-matrix organisation, profibrotic TGF-β signalling, macrophage recruitment, and interferon-driven immune activation—hallmarks of advanced MASH pathology [24, 25]. The fact that MASH-GA reproduces this established Obese → NAFLD → NASH pathway progression underscores the biological reliability of the platform.

## Discussion

MASH-GA addresses a critical unmet need for an integrated, cross-species transcriptomic resource dedicated to MASH. Compared with existing tools, MASH-GA uniquely combines rigorous manual curation, multiattribute querying, coexpression analytics, and harmonized human–mouse datasets. Our COL1A1 case study demonstrates the platform’s ability to recapitulate known fibrosis biology, validate markers across cohorts, and map expression trajectories onto murine models, thus facilitating translational research. Transition analysis further highlights the capacity of MASH-GA to dissect molecular programmes across disease stages. In early obese livers, transcriptional changes are dominated by lipogenic rewiring without overt fibrotic signatures. NAFLD is associated with the emergence of mild inflammatory responses. In contrast, NASH is characterized by pronounced immune activation and fibrotic remodelling. Current limitations include the primary focus on bulk transcriptomic data. Future development will incorporate single-cell RNA sequencing, epigenomic, proteomic, and mutation or copy number variation (CNV) datasets. In addition, automated user-data upload and real-time meta-analysis pipelines will enhance community engagement. Finally, incorporation of drug response signatures could accelerate target prioritization and repurposing efforts.

### Update of MASH–GA

MASH-GA will be regularly updated to incorporate the latest transcriptomic data and ensure the platform remains current and relevant. The current version of MASH-GA includes only datasets related to bulk RNA expression. However, other genomic and multiomics features—such as somatic mutations, CNVs, epigenomics, proteomics, and single-cell transcriptomics—are equally important for a comprehensive understanding of MASH pathogenesis. In future versions, we aim to expand MASH-GA by integrating these additional data types to provide a more complete and up-to-date resource for the research community.

## Data Availability

MASH-GA is freely accessible at https://mashga.com. All processed expression matrices, metadata and comparison Tables can be downloaded without registration.
